# Improving Cyclability of Lithium Metal Anode via Constructing Atomic Interlamellar Ion Channel for Lithium Sulfur Battery

**DOI:** 10.1186/s11671-021-03508-z

**Published:** 2021-03-23

**Authors:** Mao Yang, Nan Jue, Yuanfu Chen, Yong Wang

**Affiliations:** State Key Laboratory of Electronic Thin Films and Integrated Devices, University of Electronic Science and Technology of China, Chengdu, 610054 China

**Keywords:** Lithium sulfur batteries, Lithium dendrites, Interlamellar ion channel, Layered silicate clay, Nano composites

## Abstract

Uniform migration of lithium (Li) ions between the separator and the lithium anode is critical for achieving good quality Li deposition, which is of much significance for lithium metal battery operation, especially for Li–sulfur (Li–S) batteries. Commercial separators such as polypropylene or polyethylene can be prepared by wet or dry processes, but they can indeed cause plentiful porosities, resulting in the uneven Li ion stripping/plating and finally the formation of Li dendrites. Thence, we constructed an atomic interlamellar ion channel by introducing the layered montmorillonite on the surface of the separator to guide Li ion flux and achieved stable Li deposition. The atomic interlamellar ion channel with a spacing of 1.4 nm showed strong absorption capacity for electrolytes and reserved capacity for Li ions, thus promoting rapid transfer of Li ions and resulting in even Li ion deposition at the anode. When assembled with the proposed separator, the Coulombic efficiency of Li||Cu batteries was 98.2% after 200 cycles and stable plating/stripping even after 800 h was achieved for the Li||Li symmetric batteries. Importantly, the proposed separator allows 140% specific capacity increase after 190 cycles as employing the Li–S batteries.

**Supplementary Information:**

The online version contains supplementary material available at 10.1186/s11671-021-03508-z.

## Introduction

With ever-increasing demand for high-performance electronic applications such as electric vehicles and portable systems, the research focused on energy storage devices with high energy density and long cycle life has received extensive attention [1–3]. Specifically, lithium-metal batteries (LMBs) such as lithium sulfur (Li–S) batteries are able to deliver excellent energy storage performance owing to high energy density, prospective for practical applications [4–6]. Notably, Li metal has been employed as a promising anode material, since it has high theoretical storage capacity (~ 3860 mAh g^−1^), low standard potential (− 3.04 V v.s. the standard hydrogen electrode) and light weight density (0.53 g cm^−3^). Nevertheless, the existence of irregular pores in commercial separators can lead to poor quality of deposited lithium, which can result in dendritic formation and consuming more lithium metals and electrolytes during the repeated plating/stripping processes [7, 8].

Consequently, Li dendrites could form the “dead” Li metal when they are easily broken away from the conductive collector, resulting in low Coulomb efficiency (CE) and irreversible capacity loss [9, 10]. Additionally, Li dendrites could pierce the separator and thus cause short circuit of LMBs, further leading to thermal runaway, fire and even possible explosion of rechargeable batteries [11, 12]. Because of such obstacles, the use of LMBs in rechargeable batteries has been indeed limited in the past 20 years. Therefore, preventing the formation of lithium dendrites can be an effective approach to fully exploit promising features of LMBs [13]. Recently, researchers have proposed various methods to resolve the above issue, including optimizing electrolyte composition [5, 14], constructing the artificial solid electrolyte interphase (SEI) layer on Li metal anode [15], developing the three-dimensional composite Li anode [16], and modifying the collector [17, 18]. Although those strategies were designed to stabilize the SEI layer and/or reduce the effective applied current density of lithium metals, they were primarily focused on lithium metals and electrolytes. To date, only a few works have been conducted to address or mitigate the dendrite challenges by modifying the separator [19]. Evidently, regulating the separator can be a novel and feasible method to inhibit the formation of lithium dendrite.

Among the components of LMBs, the separator not only plays a key role to segment anode and cathode electrodes for avoiding short circuit, but also directly affects the performance of batteries via authorizing Li ion migration [9, 20, 21]. Thence, it has been reported that simple modification of the separator using semi-solid polymer electrolyte interlayer [22], graphene [23] or high modulus surface coating [24] can effectively prevent the formation of dendrites and thus improve LMBs performance. Amongst the previously reported approaches, however, the barrier layers were thick (> 10 µm) and had high mass loading (several milligrams), which can inevitably impede the rapid diffusion of Li ions and reduce energy density of LIBs. In addition, most LMBs using those functional separators can only cycle with low current densities, for instance, smaller than 2 mA cm^−2^. To improve the critical current density of LMBs, the addition of inorganic particles within the separator to improve the porous structure and increase critical current density can be another effective method. However, uneven pore distribution in the separator can generally lead to disordered diffusion of Li ions during the plating/strip process, leading to the uneven deposition of Li ions and the formation of Li dendrites [7]. Therefore, the separator microstructure with a uniform lithium transfer channel is greatly beneficial to eliminate the encountered problem of dendrite during the charge/discharge processes.

In this work, aiming to guide the migration of Li ions evenly through the separator, a Li-based montmorillonite (Li-MMT) modified composite separator is fabricated via constructing atomic interlamellar ion channels on the PP separator. The as-prepared separator embedded with interlamellar spacing (~ 1.4 nm) provides abundant active sites for Li ion diffusion and electrolyte wetting [25]. Thus, the modified separator is allowed to achieve uniform deposition of Li ions on the Li anode by unifying the direction of Li flows, which can effectively eliminate the Li dendrite issues in the charge/discharge processes. As a result, the Li-MMT separator enables the Li||Cu batteries to deliver 98.2% CE even after 200 cycles and ensures the Li||Li symmetric batteries to actualize stable plating/stripping over 800 h at 1 mA cm^−2^ with a capacity of 1 mAh cm^−2^. Moreover, the batteries with Li-MMT@PP separators also deliver good cycle stability with 140% specific capacity increased as compared to PP separators after 190 cycles at 0.5 mA cm^−2^ with sulfur loading of 1.5 mg cm^−2^.

## Experimental Methods

### Materials and Preparations

Montmorillonite (MMT), polyvinylidene fluoride (PVDF), and lithium hydroxide (LiOH) were purchased from Aladdin. The N-methyl pyrrolidone (NMP) and sulfuric acid (H_2_SO_3_) were obtained from Sinopharm Chemical Reagent Co., Ltd. Sulfur powder (S) and acetylene black (denoted as C powder) were purchased from Alfa Aesar. Celgard 2500 was used as separator. The Li-MMT powder was prepared via cation exchange. Typically, 0.2 M H_2_SO_3_ solution was used to covert the cations within the interlayer of MMT to ions and then LiOH solution was used to make the solution at PH = 7 as well as covert the hydrogen ions to the Li ions. Freeze-drying technology was used to collect the Li-MMT powder. For the preparation of Li-MMT@PP separator, only one side of the separator was coated the Li-MMT slurry that Li-MMT and PVDF powder with mass ratio 9:1 were uniform dispersed in the NMP solution and the average mass loading of Li-MMT is only ~ 0.15 mg cm^−2^.

### Characterization

X-ray diffraction (XRD) spectrum using a UltimaIV diffractometer with CuKα1 radiation (λ = 1.4506 Å) was employed to investigate the crystal structure of Li-MMT powder. High-resolution transmission electron microscope (HRTEM) was used to observe the interlayer of Li-MMT and the scanning electron microscope (SEM, FEI NANOSEI 450) was used to analyze the surface morphologies.

### Electrochemical Measurements

For the Li||Cu and Li||Li battery tests, typically, the Cu foils were firstly washed with deionized water and ethanol three times to remove the possible impurities. Then, the lithium foil was cut into circles with area of 1 cm^−2^ to use as the Li sources. The electrolyte was 1 M bistrifluoromethanesulfonimide lithium salt (LiTFSI) in a mixture of 1,3-dioxacyclopentane (DOL) and 1,2-dimethoxyethane (DME) (1:1 v/v) with 2 wt% lithium nitrate (LiNO_3_) as additive. For the Li–S battery tests, the S cathode was prepared via our previous method that The C and S powder were mixed and heated at 155 ℃ for 24 h with a mass ratio of 8:2 [26]. And then the powders of C/S composites, C and PVDF with mass ratio of 8:1:1 were uniformly dispersed in the NMP solution to prepare the sulfur electrode. The average sulfur loading is 1.5 mg cm^−2^ which was coated on the carbon coated aluminum foil. The batteries were assembled via stainless steel coin battery (CR2025) in an argon-filled glove box. Li foil was used as anode. 20 uL electrolyte was used to wet the lithium anode and additional 20 µL was used to wet the separator and cathode. Before testing, the assembled Li–S batteries was rest 12 h and then 0.2 mA cm^−2^ with 5 cycles was used to active the battery performance. The electrochemical test system was CT2001A battery test system (LAND Electronic Co., China). The cut-off voltage was 1.7–2.7 V. Electrochemical impedance spectroscopy (EIS) was tested by Electrochemical workstation (CHI660E, Chenhua Instruments Co., China).

## Results and Discussion

**Fig. 1:**
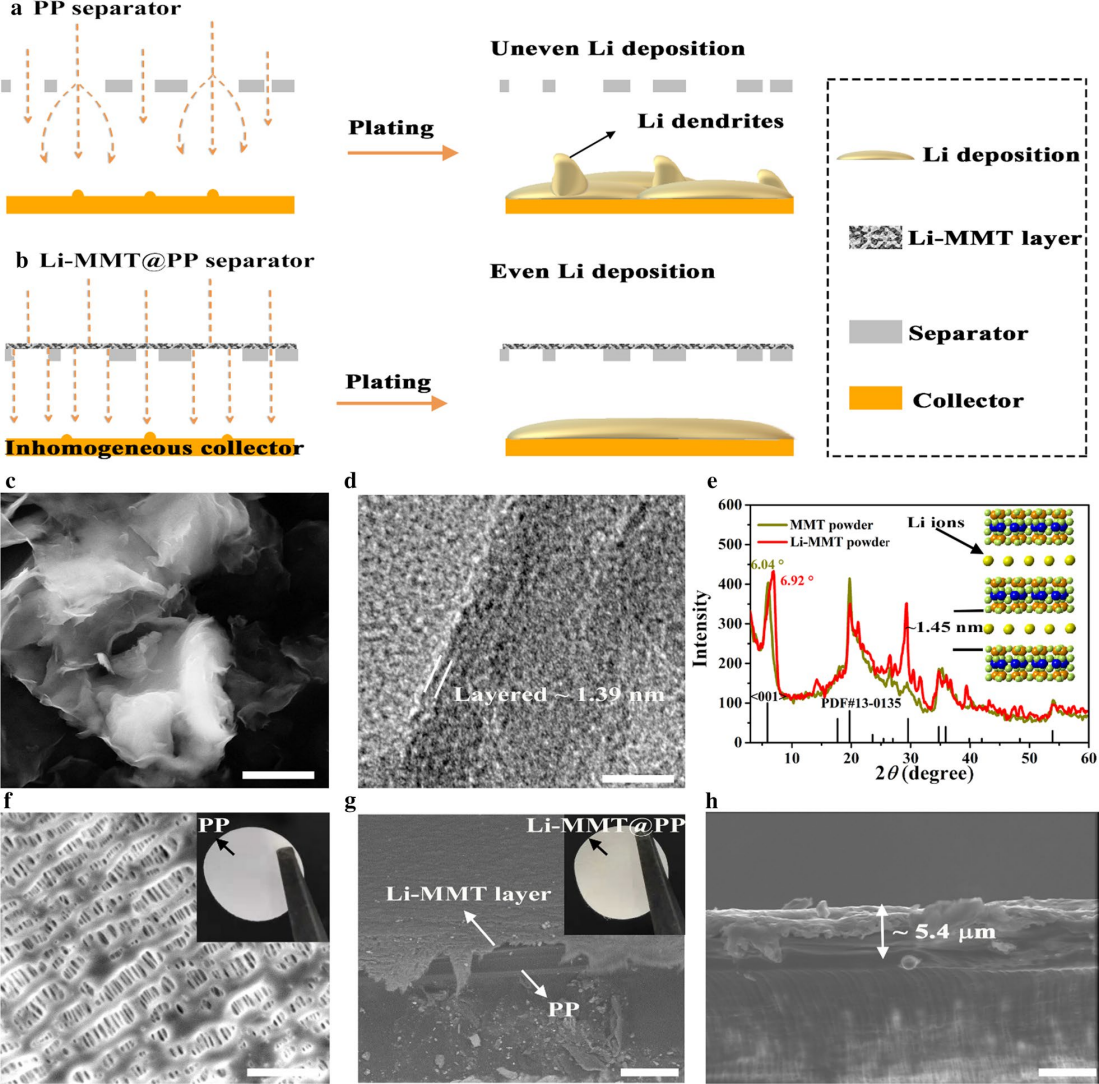
Preparation and characterizations of Li-MMT powders and Li-MMT@PP separator. **a**, **b** Schematics of design concepts with different separators. **c** SEM image of Li-MMT. **d** HRTEM image of Li-MMT. **e** XRD spectrum. **f** SEM image of PP separator, the inserted optical image is PP separator. **g** SEM image of Li-MMT@PP separator and **h** corresponding cross profile, the inserted optical image in **g** is Li-MMT@PP separator. Scar bar: **c** 1.5 µm, **d** 5 nm, **f** 2.5 µm, **g** 25 µm, **h** 5 µm

To illustrate the lithium ion flux across the commercial PP separator, the schematics are shown in Fig. 1a, b, in which the ~ 5 µm Li-MMT layer was uniformly coated on the PP separator to guide the flux of Li ions. It is well known that the commercial PP separator is typically prepared by the dry or wet processes, and then the separator is stretched to generate plentiful voids to allow Li ion pass. However, the commercial PP separator shows higgledy-piggledy paths and arbitrarily stacked pores (Fig. 1a), thus can fail to realize uniform migration of Li ions and finally cause Li dendrites. Therefore, atomic ion channel Li-MMT was employed as the modulator to guide even flow of Li ions (Fig. 1b) and achieve uniform Li deposition. The crystal structure of MMT is typically composed of negatively charged layers (NCLs) separated by interlayer space (> 1 nm), which hosts the exchangeable cation ions, such as Li^+^, Na^+^, Mg^2+^, Ca^2+^, etc. Therefore, cation exchange method is necessary to covert the host cations to Li ions [25]. The basic structure of NCLs is a typical T-O-T layer, where "T" stands for the tetrahedral sheet and "O" is for the octahedral sheet [25]. With the unique interlayer structure of Li-MMT, the electrolyte can effectively penetrate into the Li-MMT layer, resulting in the unimpeded transport of Li ions, thus achieving the efficient diffusion of ions [7, 25]. The morphology of Li-MMT is shown in Fig. 1c, d that clearly shows typical 3D nanosheet structure with closely and arbitrarily stacked architecture. According to the HRTEM image, the layered structure of Li-MMT can be observed and shows an interlayer space of ~ 1.39 nm.

The precise measurement of interlayer space of Li-MMT is shown in Fig. 1e. Raw MMT with the indeterminate cations in its interlayer presents a peak around 6.04°. After ion exchange, the peak located at 6.92° is able to confirm the change of indeterminate cations to the Li ions. Since the cations within raw MMT are highly variable in size and distribution while the Li ions are smaller size than other cations [25], causing the interlayer distance decreases gradually. According to Bragg's law, the interlayer spacing of Li-MMT can be estimated to be ~ 1.4 nm, which can provide a wide channel for Li ion transport and electrolyte wetting. The porous morphology of PP separator is presented in Fig. 1f. After coating the Li-MMT layer, it can be found the porosity of Li-MMT@PP separator is significantly decreased (Fig. 1g), beneficial for the regular ion movement. In this work, the Li-MMT slurry was coated using a coating machine, which shows potential for large-scale production. The coated thickness is only 5 µm (Fig. 1h) with negligible mass increase.

**Fig. 2:**
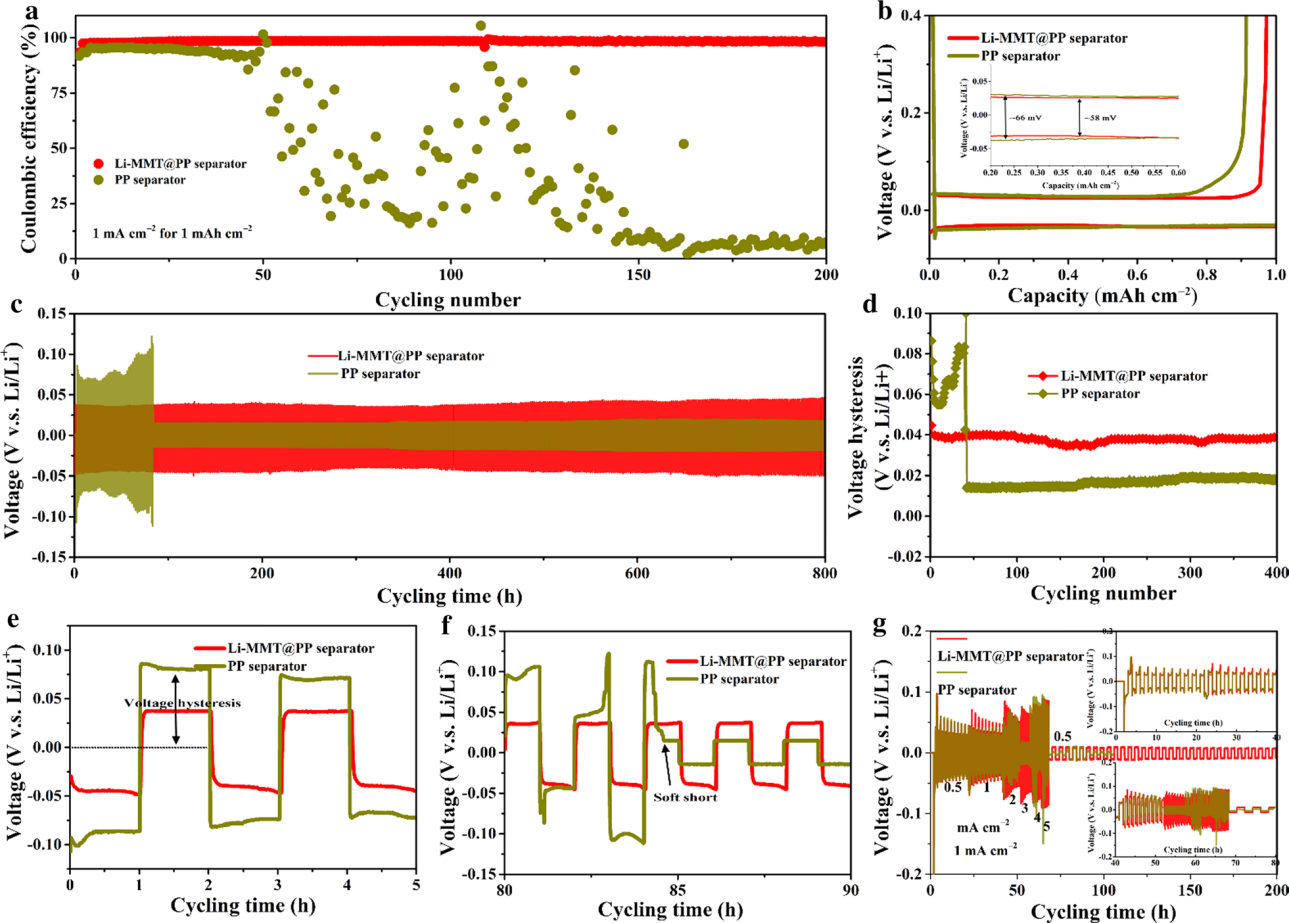
Electrochemical performances of Li||Cu and Li||Li symmetric batteries. **a** CE curves and **b** corresponding voltage curves. **c** Voltage–time profiles of the Li||Li symmetric batteries using Li-MMT@PP or PP separator at 1 mA cm^−2^ with a capacity of 1 mAh cm^−2^. **d** Voltage hysteresis of Li||Li symmetric batteries. **e**, **f** The partial enlargement profiles of **c**. **g** The rate performances of Li||Li symmetric batteries

Benefited from the aforementioned atomic interlamellar ion channel, the Li-MMT@PP separator is effective for regulating the Li deposition and suppressing the Li dendrite growth at an atomic scale via guiding the Li ion flux. The Brunner–Emme–Teller (BET) measurements show the pore size distribution of Li-MMT powder within the scope of 1–3 nm (Additional file 1: Fig. S1). As shown in Fig. 2a, the Li||Cu battery was employed to study the CE. It is found that the Li-MMT@PP separator can deliver the Li||Cu battery with high CE and excellent stability even over 200 cycles at the current density of 1 mA cm^−2^ with a capacity of 1 mAh cm^−2^. During the tests, it can be observed that all of the CE present an upward trend in the first 5 cycles, caused by the surface passivation of Li deposition. However, higher average CE in the first 5 cycles of Li-MMT@PP separator highlights the advantages that the deposited Li metal suffers lower side reaction with the liquid electrolyte as coupled with Li-MMT@PP separator. With the reduplicative plating/stripping, the shortcoming of PP separator is gradually exposed that the assembled Li||Cu battery only endures ~ 50 cycles and its CE decreases sharply to 60% and almost to zero after 150 cycles. On the contrary, the CE of Li||Cu battery assembled with Li-MMT@PP separator still delivers stable cycles with lower over-potential (Fig. 2b) and the battery still maintains 98.2% CE after 200 cycles, indicating the deposited Li metal is more uniform and no lithium dendrite is produced after the regulation of Li-MMT layer.

To further investigate the advantages of the Li-MMT@PP separator in the cycling stability of Li metal anodes, symmetric Li||Li batteries with various separators are also fabricated. As shown in Fig. 2c, when the cycling capacity is 1 mAh cm^−2^ at a current density of 1 mA cm^−2^, the battery with the Li-MMT@PP separator delivers an excellent cycling stability with stable voltage plateaus over 400 cycles (900 h) (Fig. 2d). In sharp contrast, the battery with the PP separator exhibits strong voltage hysteresis in the initial stages. The overpotential is almost two times than that of Li-MMT@PP separator (Fig. 2e). After Li plating/stripping over 84 h, a sudden voltage drop is observed for the battery with the PP separator (Fig. 2f), which can be ascribed to the electrical connection between the electrodes, resulting in the “soft short”. Therefore, the rate performances of Li symmetrical battery were further used to assess the current density in suppressing the Li dendrites. As shown in Fig. 2g, the Li-MMT@PP separator under the current density even as high as 5 mA cm^−2^ still exhibits normal plating/striping behavers. The PP separator appears significant voltage fluctuations as the current density closing to 3 mA cm^−2^. Especially when the current density is increased to 5 mA cm^−2^, the voltage becomes extremely unstable, indicating the Li anode surface suffers serious Li dendrites. As compared with previous works (Additional file 1: Table S1), the Li-MMT modified separator shows competitive advantages to effectively suppress the Li dendrites.

**Fig. 3:**
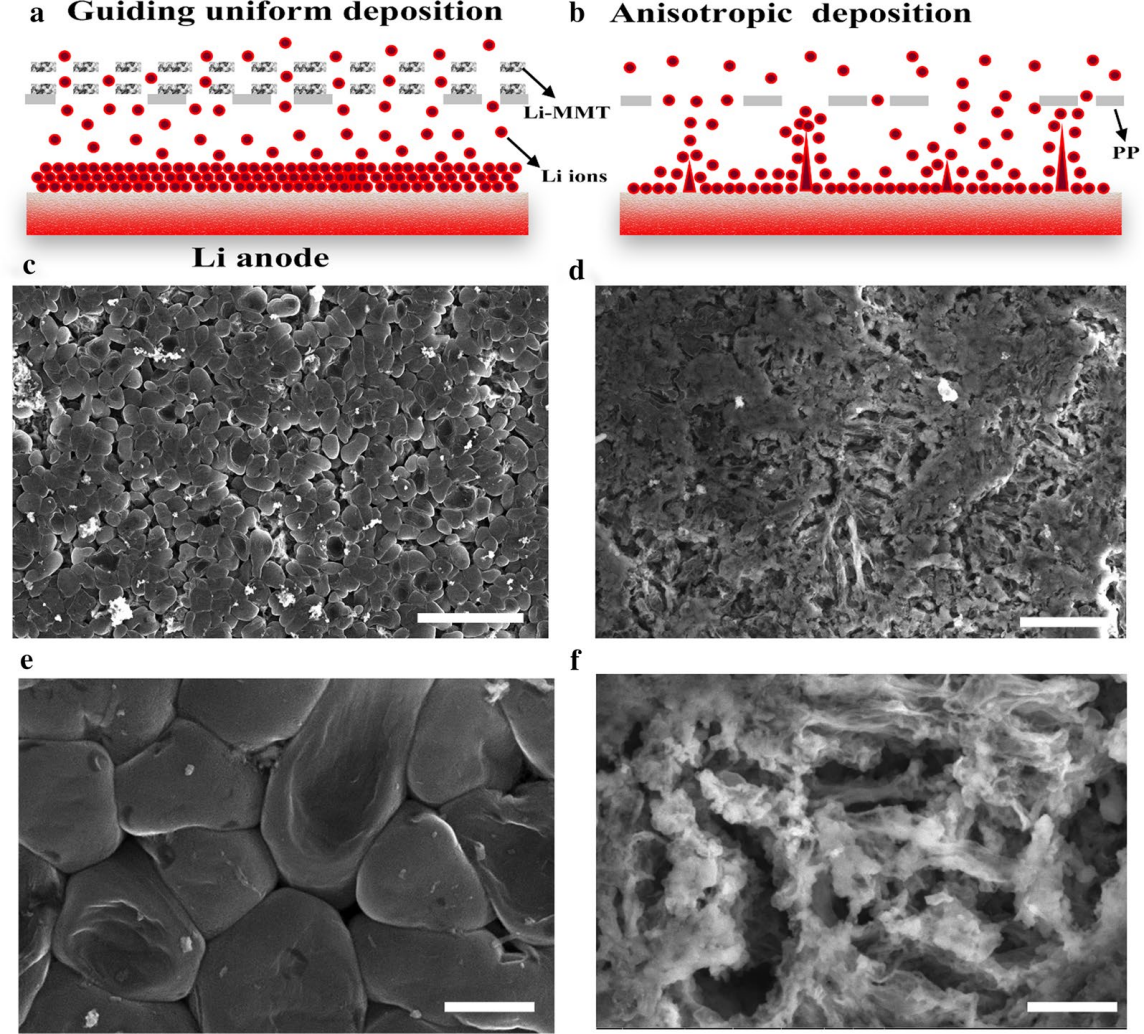
SEM images of Li anode coupled with Li-MMT@PP or PP separator after 20 cycles at 1 mA cm^−2^ with a capacity of 1 mAh cm^−2^. **a**, **b** Mechanism illustration of Li-MMT@PP or PP separators. **c**, **e** Li-MMT@PP separator. **d**, **f** PP separator. Scale bars: **c** 25 µm, **d** 10 µm, **e**, **f** 2.5 µm

The correlation of Li ions across the separator before and after coating the Li-MMT layer is proposed in Fig. 3a. After cation exchange, the interlayer of Li-MMT provides the active site for Li. The interlayer spacing of 1.4 nm serves as a unique Li ion channel to allow the regular flux of Li ions during the plating/stripping processes. However, for the PP separator, the higgledy-piggledy paths (Fig. 3b) and arbitrarily stacked pores will fail to allow the uniform migration of Li ions as across the separator, leading to the heterogeneous deposition of Li ions in the electrochemical processes, and causing the formation of lithium dendrites. Thus, the morphologies of Li metal anodes after 20 cycles are investigated to further clarify the effect of Li-MMT@PP separator on the suppression of Li dendrites. As shown in Fig. 3c, e, after coating the Li-MMT layer, uniform and dense Li deposition is realized and no formation of Li dendrite is observed on the anode surface even after 20 cycles. Importantly, the Li metal anode still retains a relatively dense and compact structure with dendrite-free surface, highlighting the advantages of Li-MMT layer for favorable dendrite-free Li plating/stripping behavior. However, for the cell with PP separator, the Li metal anode displays obvious wire-shaped Li dendrites after cycles (Fig. 3d), and loosely stacks mossy Li with a highly porous structure (Fig. 3f).

**Fig. 4:**
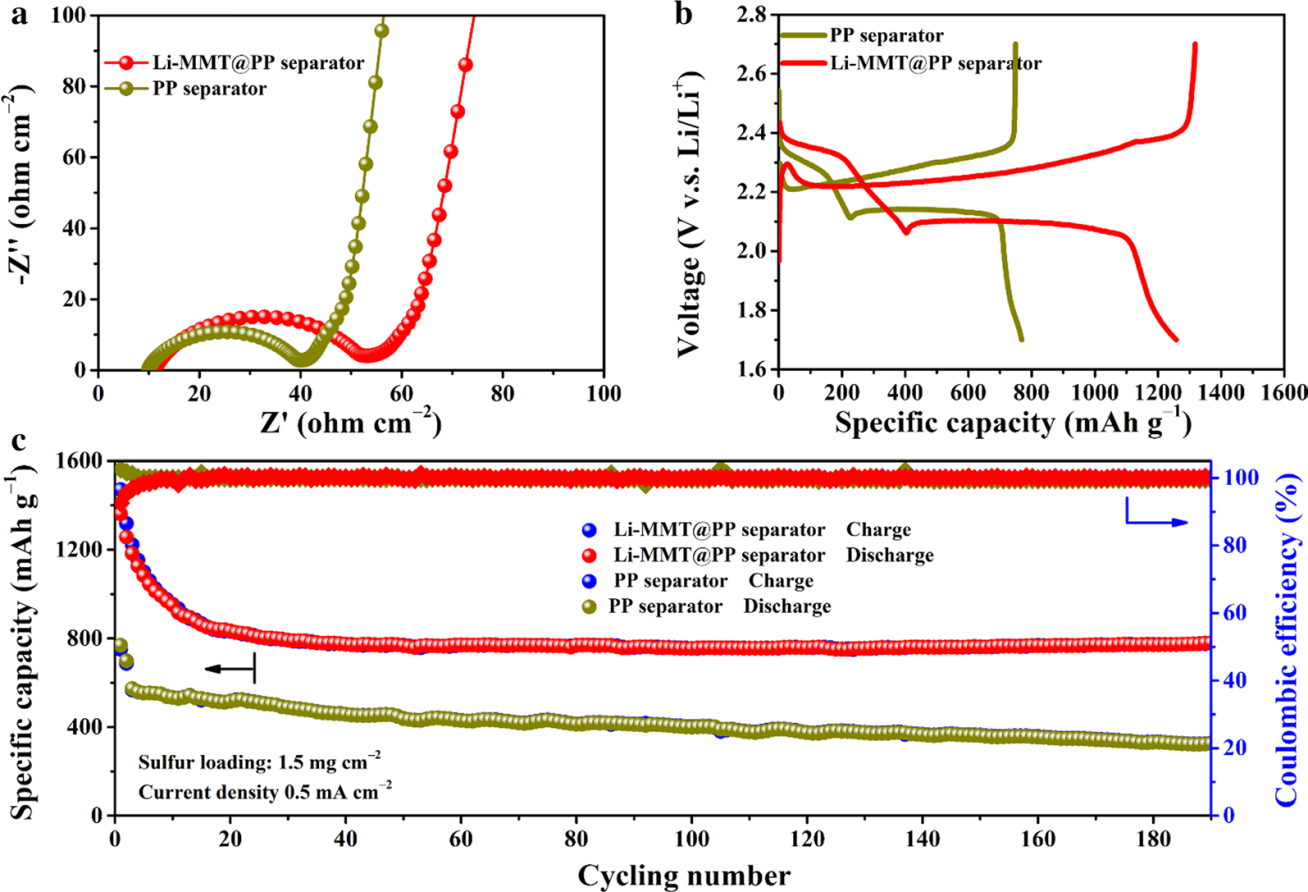
Electrochemical performance of Li–S batteries with different separator. **a** EIS results. **b** The charge/discharge plateaus with Li-MMT@PP or PP separator. **c** Long-term cycling performance at 0.5 mA cm^−2^ with sulfur loading of 1.5 mg cm^−2^

To demonstrate the potential of Li-MMT@PP separator in the practical application of Li metal batteries, the S cathode with S loading of 1.5 mg cm^−2^ was employed as the electrode. The electrochemical interface assembled with different separators was investigated by the electrochemical impedance spectroscopy (EIS) measurement. As shown in Fig. 4a, typically, all of the separators display depressed semicircles at high frequencies, which are correspond to the interfacial charge transfer resistance. Although we can see that the charge transfer resistance of the battery assembled with Li-MMT@PP separator is slightly greater than that of PP separator, the battery's performance was not affected after low current density activation, which has been claimed in the experimental section. During the low frequency regions, the sloping lines present the lithium ion diffusion within the active materials. Figure 4b shows the voltage plateaus of C/S composite cathode assembled with Li-MMT@PP or PP separators between 1.7 and 2.8 V (V.S. Li/Li^+^). The cyclic voltammetry (CV) tests were conducted and presented in Additional file 1: Fig. S2. Although the larger concentration of polysulfides only generates slightly larger concentration polarization than the PP separator, the peak area of Li-MMT@PP separator is much larger than that of PP separator, indicating that more polysulfides are generated when using the Li-MMT coating layer. According to the reaction mechanisms of S cathode, the Li–S battery exhibits two typically plateaus during the charging/discharging processes. In the first stage before the knee point, the Li-MMT@PP separator delivers a high discharge capacity of ~ 400 mAh g^−1^ with negligible voltage hysteresis. However, for the PP separator, only ~ 210 mAh g^−1^ capacity is observed, indicating that partially released long-chain polysulfides (especially for the Li_2_S_8_) are not involved in the subsequent redox reaction to contribute the capacity. The higher discharge capacity during the first plateau implies the Li-MMT layer can effective avoid the shuttle of soluble long-chain polysulfides to the Li anode surface. At the second conversion steps, obviously, for the PP separator, small amounts of short-chain polysulfides are formed due to the existence of shuttle effect within the ether-based electrolyte, which has been confirmed by our previously work [26]. In contrast, the Li-MMT@PP separator is rational designed that the Li-MMT surface has strong anchoring ability for polysulfides to avoid the shuttle of polysulfides [25]. The excellent adsorption properties ensure that the polysulfides are prevented to spread the Li anode surface and passivate the Li surface, thus allowing Li–S battery assembled with Li-MMT@PP separator has a high discharge capacity of 1283 mAh g^−1^. Long-term cycles with good stability are the primary goals for the commercial batteries. The long-term cyclability of Li-MMT@PP separators is shown in Fig. 4c. In the early 20 cycles, it can be observed that the capacities of Li-MMT@PP and PP separator show a typical decreasing trend. This is because, in the early discharge process, plentiful polysulfides would precipitate from the inner of C/S cathode material and deposit on the surface of the cathode material [26], resulting in the loss of capacity. However, after stabilizing the lithium metal anode, the benefits of Li-MMT@PP separator are emerged that the retention of discharge capacity maintains 100% during the subsequent cycles and the CE is also 100%.

## Conclusions

In summary, the interatomic ion channel (Li-MMT) was constructed on the porous PP separator to modulate the Li ion flux and then guide the even deposition of Li ion on the Li anode during the electroplating/stripping. Due to the wide interlayer space (~ 1.4 nm) of Li-MMT, the Li-MMT@PP separator greatly ensures the cyclability of Li metal anode by unifying the flow direction of lithium ions, resulting in the uniform deposition of Li ions on the anode surface, thus forming a dendritic free lithium anode. As assembled with the Li-MMT@PP separator, the Li–S battery exhibits a remarkable reversible capacity of 776 mAh g^−1^ (almost 1.4 times larger than PP separator) with a 100% CE after 190 cycles at the current density of 0.5 mA cm^−2^ with the sulfur loading of 1.5 mg cm^−2^.

## Supplementary Information

**Additional file 1: Table S1.** The comparison of Li-MMT@PP separator with previously reported functional separator. **Fig. S1.** BET results of Li-MMT powder. **Fig. S2.** Cyclic voltammograms of PP and Li-MMT@PP separator, recorded at 0.1 mV/s.

## Data Availability

All data are fully available without restriction.

## Abbreviations

Li: Lithium
Li–S: Lithium sulfur
PP: Polypropylene
PE: Polyethylene
MMT: Montmorillonite
CE: Coulombic efficiency
LMBs: Lithium-metal batteries
SEI: Solid electrolyte interphase
Li-MMT: Li-based montmorillonite
PVDF: Polyvinylidene fluoride
LiOH: Lithium hydroxide
NMP: N-methyl pyrrolidone
H_2_SO_3_: Sulfuric acid
C: Acetylene black
XRD: X-ray diffraction
HRTEM: High-resolution transmission electron microscope
LiTFSI: Bistrifluoromethanesulfonimide lithium salt
DOL: 1,3-Dioxacyclopentane
DME: 1,2-Dimethoxyethane
LiNO_3_: Lithium nitrate
EIS: Electrochemical impedance spectroscopy
NCLs: Negatively charged layers
BET: Brunner–Emmet–Teller
CV: Cyclic voltammetry
