# Supplementary material for: Improving Cyclability of Lithium Metal Anode via Constructing Atomic Interlamellar Ion Channel for Lithium Sulfur Battery
Source: Nanoscale Res Lett. 2021 Mar 23;16:52. doi: 10.1186/s11671-021-03508-z (PMC7988023; doi:10.1186/s11671-021-03508-z)
Supplement: Supplementary file 1 — Additional file 1: Table S1. The comparison of Li-MMT@PP separator with previously reported functional separator. Fig. S1. BET results of Li-MMT powder. Fig. S2. Cyclic voltammograms of PP and Li-MMT@PP separator, recorded at 0.1 mV/s. [file 11671_2021_3508_MOESM1_ESM.docx]

**Supplementary Information**

**Improving Cyclability of Lithium Metal Anode via Constructing Atomic Interlamellar Ion Channel for Lithium Sulfur Battery**

Mao Yang^1^, Nan Jue^1^, Yuanfu Chen^1^, Yong Wang^1,*^

^1^State Key Laboratory of Electronic Thin Films and Integrated Devices, University of Electronic Science and Technology of China, Chengdu 610054, China.

*Corresponding author: ywang@uestc.edu.cn.

Table S1. The comparison of Li-MMT@PP separator with previously reported functional separator.

| Separators | Coating  thickness  (μm) | Coated  loading  (mg/cm^2^) | Current  density(max)  (mA/cm^2^) | Reported cycling  time (h) (current density mA/cm^2^) | | Ref. |
| --- | --- | --- | --- | --- | --- | --- |
| Li-MMT/PVDF-HFP | \ | \ | 1 | 300 (0.5) | [1] | |
| Si-PP | 2 | 0.2 | 2 | 1000 (0.5) | [2] | |
| CTS-PEO-PTEGDMA@Celgard | 0.025 | \ | 6 | 200 (1) | [3] | |
| MH/PP/MH | 5 | \ | 1 | 400 (1) | [4] | |
| BNxGry/PP | 10 | \ | \ | 1000 (\) | [5] | |
| **Li-MMT@PP** | **5.4** | **0.15** | **5** | **800** | **This work** | |

Notes:Li-MMT/PVDF-HFP: Li-montmorillonite/poly(vinylidene fluoride-co-hexafluoropropylene) (PVDF-HFP); Si-PP: Silicon (Si) coating onto the polypropylene (PP) separator; CTS-PEO-PTEGDMA@Celgard: chitosan (CTS), polyethylene oxide (PEO), and poly(triethylene glycol dimethacrylate) (PTEGDMA); MH/PP/MH separators: Mg(OH)_2_ nanoflake/PP/Mg(OH)_2_ nanoflake; BNxGry/PP: Boron nitride-graphene (BNxGry) layer coated on one side of polypropylene (PP) membrane.


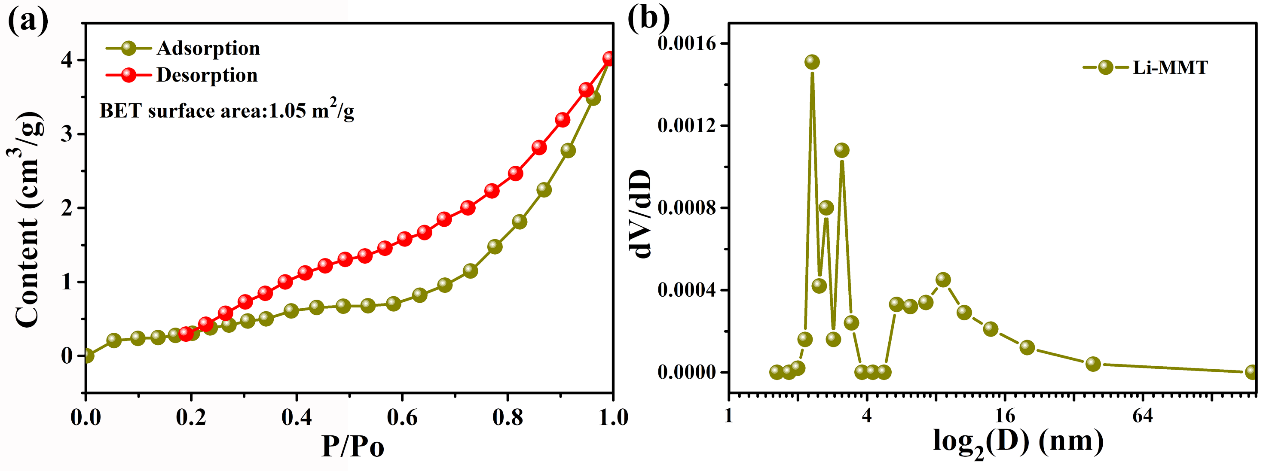


Fig. S1. BET results of Li-MMT powder.





Fig. S2. Cyclic voltammograms of PP and Li-MMT@PP separator, recorded at 0.1 mV/s.

**References**

1. Zhao J, Chen D, Boateng B, et al (2020) Atomic interlamellar ion path in polymeric separator enables long-life and dendrite-free anode in lithium ion batteries. J Power Sources 451:227773. https://doi.org/10.1016/j.jpowsour.2020.227773

2. Chen X, Zhang R, Zhao R, et al (2020) A “dendrite-eating” separator for high-areal-capacity lithium-metal batteries. Energy Storage Mater 31:181–186. https://doi.org/10.1016/j.ensm.2020.06.037

3. Shen L, Liu X, Dong J, et al (2020) Functional lithiophilic polymer modified separator for dendrite-free and pulverization-free lithium metal batteries. J Energy Chem 52:262–268. <https://doi.org/10.1016/j.jechem.2020.04.058>

4. Lim JH, Yang MH, Lee JW (2020) Bifunctional carbon monofluoride (CFx) coating on a separator for lithium-metal batteries with enhanced cycling stability. J Electroanal Chem 878:114586. https://doi.org/10.1016/j.jelechem.2020.114586

5. Rodriguez JR, Kim PJ, Kim K, et al (2021) Engineered heat dissipation and current distribution boron nitride-graphene layer coated on polypropylene separator for high performance lithium metal battery. J Colloid Interface Sci 583:362–370. https://doi.org/10.1016/j.jcis.2020.09.009
